# The pioneers of nephrology – Professor Natalia Tomilina: courage, passion and humanism in medicine

**DOI:** 10.1186/s12882-019-1252-y

**Published:** 2019-03-19

**Authors:** Giorgina B. Piccoli, Gilberto Richiero, Hayley Henderson

**Affiliations:** 1 Dipartimento di Scienze Cliniche e Biologiche, Università di Torino, Turin, Italy; 2 Nephrologie, Centre Hospitalier du Mans, 198 Avenue Roubillard, 72000 Le Mans, France; 3 Via Susa 47 Chiusa san Michele 10050, Torino, Italy; 4 BioMed Central, The Campus, 4 Crinan St, London, N1 9XW UK

## Abstract

Listening to the interview of Natalia Tomilina is an inspiring experience, and not one reserved purely for young physicians. Within these pages, one can discover Tomilina’s determination and passion for learning that has been with her throughout her life, even during difficult and testing times. A great resolve that she developed through the teachings of her parents and her mentor, Prof. Maria Ratner.

It is clear that her ties to her cultural roots are strong, allowing her to have a greater understanding of her patients (“*the doctor has to understand the patient*”), and with this, she has developed a humanist approach to medicine. These great attributes have ensured that Tomilina’s contributions to the field of nephrology have been significant – her belief being that her discoveries in medicine belong to the patients and not to the physicians.

Those who are older will find the stories of her trials and tribulations in old Russia fascinating, as you rediscover what life was like for a female scientific researcher behind the “Iron Curtain”.

I think that, regardless of age, the nephrology community would like to join us in paying homage to a great woman, whose life tells us that changing the world is possible.

“*Prosperity is not the main point, and it is not prosperity that gives you satisfaction.”*

The interview was recorded in Prague in June 2011.

## Main body: Professor Natalia Tomilina: an interview

Both my parents were doctors, and they desperately wanted me to become an MD. When I graduated from high school, I did not really know what I wanted to do but my parents convinced me to study medicine. They were devoted to their profession and I now see that they were right in many ways. I am very thankful to them for convincing me to become a doctor because this profession indeed offers many opportunities – especially in terms of personal fulfillment. If you are interested in basic science, you can do research. If you are interested in working with patients, you can become a practitioner. However, without analyzing your results – e.g. how successful you have been in treating patients – one cannot grow professionally. Also, if you are interested in healthcare policy and organization, being a doctor allows you to also work in this field. Therefore, if a high school graduate chooses to study medicine, they will be able to pursue a professional career that will allow them to use their skills to help others.

**Fig. 1:**
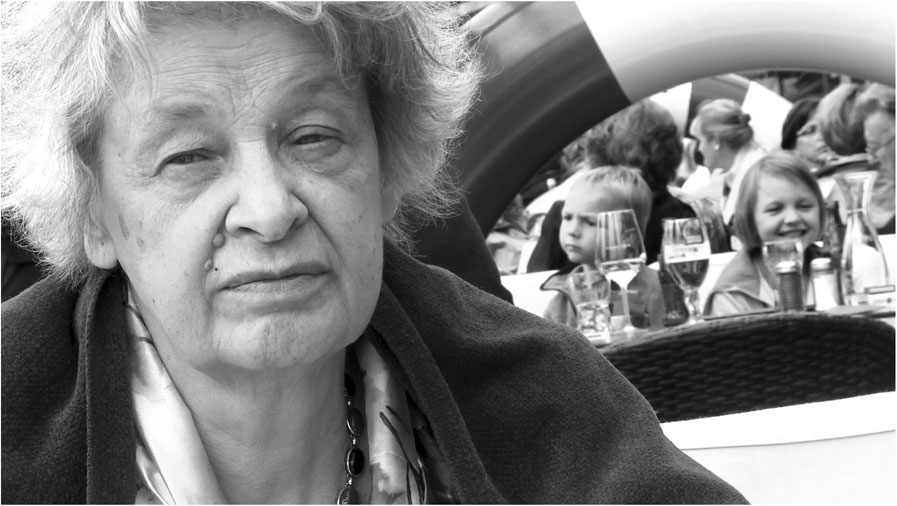
Natalia Tomilina in Prague, June 2011

For me specializing in nephrology happened by chance. After graduating from university, I worked as a general practitioner, and very soon realized that I needed something more than just routine clinical practice; I needed to grow professionally. In 1962–1963 the hospital where I worked introduced a nephrology program. It was not yet a nephrology unit, just 20 beds on the internal medicine floor for patients with kidney diseases. At the time, nephrology as a specialty was only starting to be recognized both in the Soviet Union and in other countries. I was lucky to have met Professor Maria Ratner, who invited me to work with her. I could have moved to the hospital’s research institute, but it seemed to be less interesting, so I chose nephrology and Professor Ratner became my mentor. I found it fascinating, and I have continued to be fascinated by nephrology all my life (Fig. 1).

I’ve worked in many fields of nephrology and it is difficult to decide which I like most. I did some experimental work – experimental models of glomerulonephritis, and a study of the mechanisms of nephrotic edema. After receiving my PhD, I returned to clinical practice and treated patients with glomerulonephritides, and also became interested in that. Next, by necessity, I moved to a dialysis unit and worked there for several years. I then worked in kidney transplantation for over 35 years and now my specific field of interest is post-transplant management and evaluation. About 20 years ago I founded the Moscow Nephrology Center, which was unique at the time. As the head of this center I have multiple responsibilities: our ICU for nephrology patients; the hemodialysis and peritoneal dialysis units; the transplantation unit (although we do not do surgery, we have a special unit for the recipients of renal transplants); treatment for patients with glomerulonephritis and so on. I am interested in all these fields. My special interest is transplantation, but the treatment of nephritis is no less important.

In the Soviet Union, nephrology started to be recognized as a specialized field in the early 1960s and by about 1965 the Society of Nephrology of the USSR was founded, soon after ISN [International Society of Nephrology]. It was Eugeny Tareev and Miron Woffsy, two widely-recognized professors of internal medicine, who were responsible for the development of nephrology as a specialty. Professor Tareev was the head of the Moscow University Clinic and established his own school of nephrology, while Professor Woffsy invited my teacher, Professor Ratner, to work with him and suggested she begin to specialize in renal diseases. At the time Professor Ratner had just finished her post-doctoral studies and moved from Leningrad (now Saint Petersburg) to Moscow where, as I mentioned before, the first 20-bed unit for nephrology patients had just opened. Later the Health Ministry somehow became interested in the problems of nephrology and decided there should be nephrology units in all the big cities in the USSR. Moscow’s nephrology beds became a model, and the main regulations were based on our experience. Moreover, we were the first in the country to do renal biopsies: in 1964 we did the first one ever performed in the USSR. We were also the first to use steroids to treat glomerulonephritis. Our first patient who received steroids was a child; it happened that she was the daughter of one of our doctors. The girl had a severe nephrotic syndrome caused by minimal change disease and professor Ratner successfully treated her. This also happened in the early 1960s and was the first case of its kind in the USSR.

I would like to emphasize that during the Cold War period we had little contact with the rest of the world and therefore nobody in the West knew what was going on in Soviet nephrology. Even when something really important happened – some discoveries in the field of nephrology were made – our colleagues in Europe and the US had no chance to learn about it and acknowledge it. Professor Ratner, for example, was not able to publish her studies in international journals (she published only in Soviet medical journals), and nobody outside the USSR knew about her work. Moreover, some of her discoveries were independently re-discovered by other investigators. I mention this just to point out that we were developing rapidly, in the step with countries in the West.

Professor Ratner had me read all the international textbooks. One was a huge book by Jan Brod, which I literally had to learn by heart. There were also other books, which I, and others, were told to read. At the same time, however, nephrology was not taught in medical schools and people had to study the subject on their own, and we did. We had, and still have, access to the medical literature. We have public libraries where one can get everything. The literature was available and we used to visit the library near our hospital regularly. At the time it was called the Leninskaya Library and while it has now been renamed, it is still the largest public library in Moscow. In both Moscow and Saint Petersburg people had free access to the medical literature. People from other cities also used these libraries. All the main medical journals were available in the libraries and we had access to them as well. In addition, every year, or every other year, special nephrology summer courses were held. In contrast to current practice, these courses lasted for two weeks, and people were able to study selected topics of nephrology in depth. These summer programs were organized by Prof. Yuri Natochin, a physiologist, so we studied renal physiology and pathophysiology and also clinical issues and our nephrologists therefore obtained a thorough grounding in their field. When Perestroyka began we had a substantial number of trained nephrologists working in clinical practice and research.

Our big problem was that the health authorities, the Health Ministry, did not really understand the seriousness of renal diseases. The Ministry saw cardiovascular diseases as the national health care system’s priority. For this reason, renal replacement therapy (both dialysis and transplantation) was underdeveloped. Even so, much important work was done: In 1957, Professor Nikolay Lopatkin first used hemodialysis for acute kindey failure treatment in Moscow and it was he and his co-workers, in cooperation with engineers that invented the artificial kidney used in the operation. In 1965, Professor Boris Petrovsky performed the first renal transplantation. However, until the 1990s, things moved very slowly. Classical nephrology developed rapidly, but dialysis and transplantation did not.

Perestroyka was a hard time for us. Particularly the financial reforms caused a shock. Many people were confused – the old system was collapsing, and a new one had not been established. This resulted in the appearance of a “lost generation”. What happened was that “old” nephrologists retired, and many talented middle-aged people emigrated. For example, some of my colleagues – renal physiologists, pathologists and clinicians, those who felt they would be able to start a new life abroad – left the country. Those who stayed, many of whom were elderly, could not work effectively because of the financial problems they had to deal with. Many young doctors went to work for pharmaceutical companies because they needed more money. This caused disruption.

During the Soviet period, the organization of professional schools and conferences was funded by the government. After Perestroyka, there was no longer any government financial support.

Not only did nephrologists feel lost; it seemed too many that there was no way out of the crisis. I’ll give you an example: It was decided that a conference should be held to celebrate the 100th anniversary of the birth of Professor Gynetsynsky, one of our most influential physiologists, in Novosibirsk, the city where he had lived and worked. Unfortunately, I was the only Moscow doctor able to go to Novosibirsk, because the others could not afford plane tickets. The conference had obtained a grant of one million rubles from the Academy of Science. This sounds like a huge amount of money but it was exactly what a return ticket from Moscow to Novosibirsk cost at the time.

The Society of Nephrology was also adrift. It seemed it would no longer be possible to find money to conduct meetings or support any kind of activities. Then unexpectedly, one day in 1994 I got a telephone call from Professor Barry Brenner. I was quite surprised and could not understand how he had obtained my telephone number. It turned out that in 1989 a friend of mine, who worked at the University of Moscow and also at MIT, had offered to give me a subscription to one of the international journals and I’d chosen *Kidney International*. When my friend phoned to arrange the gift subscription, it was suggested that I should become a member of ISN, and he paid the membership fee. Thus, I had been a member of ISN since 1989, and my telephone number was in their files. When ISN decided to impact the development of nephrology in emerging countries, the only telephone number they had in Russia was mine. Professor Brenner asked me whether we had nephrologists in Russia and I told him we did. He asked if I would be able to give a talk on Russian nephrology to ISN’s members and I said I definitely could. Then Professor Brenner proposed we organize ISN’s first course in Russia.

ISN’s presence in Russia helped us in a number of ways. For example, they taught us how to attract pharmaceutical companies as sponsors. Baxter was our first sponsor. The course was very successful, and the attendees and international speakers were satisfied. The speakers were quite surprised by the questions from the room; they did not expect such a level of knowledge. ISN decided to conduct courses like this one on a regular basis. We decided to found a new professional organization, called the Russian Dialysis Society, which continued working and collaborating with ISN despite all the problems and difficulties that arose. Now we have two organizations. The Society of Nephrology re-started its work after we showed how it could be done, while the Russian Dialysis Society works in cooperation with the Society of Nephrologists.

Professor Pierre Ronco asked me about our work in nephropathology. Until about 1990, we were active in this field, after which for about 15 years we stopped providing treatment as we lacked the necessary equipment, and even more importantly, Professor Viktor Serov, who worked with us for many years, retired, and the people who were expected to replace him failed to do so. We re-started nephropathology and with ISN’s help we have made good progress and I believe we will continue to.

I have never wanted to leave Russia. I couldn’t even imagine living in another country. Probably the main reason is that a doctor has to understand the patient – not only the signs and symptoms, but also life style, compliance and much more. I knew that in a foreign country I would never be able to really understand my patients. I know the habits and life style of the people in the country where I was born and raised; I can pick up if they will listen to me or not, follow my advice or not, etc. I know how to talk to them and which arguments to use. In a foreign country I could only formally prescribe medications and would never be satisfied with my job. For this reason I never wanted to leave; I have to work at home, where I know and understand almost everything about my patients. Many who left Russia were seeking better pay and a more prosperous life. Well, I think that a prosperous lifestyle sooner or later becomes boring. Prosperity is not the main point, and it is not prosperity that gives you satisfaction. I feel that each person should live where he or she has the most opportunity to obtain personal fulfillment.

Since 1993 I have been chief nephrologist at the Moscow Health Care Department. What does this mean? It means I was given the task to organize nephrology care in Moscow, which at the time was very poor. The population of Moscow is about 12 million inhabitants: like a small country. Many things had to be organized from scratch but we succeeded. In Moscow, compared to many regions, we have well-developed dialysis and transplantation programs, and the diagnostics of native kidney diseases improved. We managed to overcome many difficulties. Of course, we have a lot to do in the future, but at the moment we have a well-established health-care system for patients with kidney diseases. We are trying to expand our experience in other regions of Russia and although we still face many problems, we are moving ahead, and I feel satisfied.

My teacher, Professor Maria Ratner, was an outstanding person. She was devoted to medicine in general and to nephrology in particular. I believe that she made two significant discoveries in nephrology. First, she discovered renin secretion by the kidney, and although it was indeed a major discovery, it failed to receive international recognition because she published only in Soviet journals. The same discovery was subsequently made by another scientist, but priority does not matter. I trust that science is international, and I point out her discovery only to stress that Professor Ratner was a scientist by nature. Her second discovery was in the field of her life-long interest: she studied the role of the tubular-interstitial compartment in the progression of renal diseases. She was interested in interstitial damage in glomerulonephritis and studied the correlations between pathology findings and clinical data. Her results were ahead of the findings of other authors, for example those of Professor Adalbert Bohle in Germany, but again, priority does not matter. It may seem incredible that some nephrologists in the Soviet Union thought her interest in this question was ludicrous and failed to see the importance of her work.

At present, there are two schools of nephrology in Russia. As mentioned above, one was founded by Professor Tareev, while the other, to which I belong, was founded by Professor Ratner. Professor Ratner had a hard life under communism in the Soviet Union. She was not a member of the Communist Party and never joined any communist group or organizations. In addition she was a woman and she was Jewish. As a consequence, she never managed to be appointed to a top position. In fact she always worked in research groups headed by one academic or another, which had some official research topic. Thus, the work she did in nephrology was done in addition to her “real work”. She used to arrive at 5 p.m. (after the end of her working hours) to see the patients in the nephrology beds with us (and we had to wait for her every evening). She purchased prednisone at her own expense because before 1967–1968, when prednisone became available to in-patients, the hospital refused to buy it. The day she received her monthly salary she used to hand prescriptions and money to the senior nurse, and a substantial proportion of her salary was spent on the medicines we needed to treat out patients. She was very brave. When she decided to start doing renal biopsies, the hospital authorities threatened her: she was told that in case of complications criminal charges would be brought against her, but she continued all the same. She also received a warning when she was the first doctor in our country to use steroids. She played a very important role in my life, probably no less important that my Mother’s. She mothered me as a professional; she taught me scientific methodology. She was really an outstanding person and every day I ask myself how she would behave if she were in my position today.

She founded the unit of pediatric nephrology at the Institute of Pediatrics, and only in 1969 did she start to work officially in my hospital as an employee of the Research Institute of Transplantology and Artificial Organs. As the Institute of Transplantology was funded by the hospital I worked for, I also worked there. Although Professor Ratner eventually became the head of the Nephrology Unit at the Institute of Transplantology, her main interest was the treatment of glomerulonephritis until the end of her life. She died in 2000 at the age of 80.

In the Soviet Union nothing could be done without official support. Everything had to be approved officially. I mean literally everything. It was very difficult to work without government support, and sometimes just as difficult to work in spite of its support. The situation has now changed a great deal; there are still have many rules and regulations, but it’s a bit easier. In Soviet times you had to present a proposal and obtain approval from the Communist Party for everything. Can you imagine that the decision to recognize Professor Ratner as an Honored Scientist was approved first by the Communist Party’s committee at the Institute, then confirmed by the district’s Communist Party committee, then by the city’s Communist Party committee, and finally by the Central Committee? Four academics recommended her, but the final decision was up to the Communist Party. Now, I do not need to obtain approval to conduct meetings or conferences. I feel much freer, of course, and this is important.

When we started teaching our first nephrology courses, the medical technology company named Gambro became interested and proposed we organize a French-Russian program of nephrology studies. As a result, the programs were held in alternate years: we would organize ISN courses one year and a French-Russian program of studies the next. The moving spirit behind this program was Professor Jacques Chanard, with whom we worked closely with in deciding the scientific program. Gambro no longer supports this project but we have continued to invite Professor Chanard to speak, now as a member of ISN or EDTA. We have maintained a long-standing friendship and cooperation, not only with Professor Chanard, but also with Professor Phillipp Rheu and other French colleagues. In addition, when we re-established renal pathology, one of my co-workers trained at the Necker Hospital with Professor Pierre Noel, and they are still in contact.

It is not easy to talk about myself. I have many students. About forty of them now work at the City Nephrology Center. A few of them – four or five – are here, attending the EDTA Congress. They all became practitioners and some of them also do clinical research. I cannot complain. We are a very good team. My pupils and I are united by the same attitude towards our profession. We love our work, our profession; we are deeply interested in nephrology and our patients. All of my students work hard. They are very enthusiastic and there is no need to force or convince anybody. So there is a long-standing, close friendly relationship at the Center, which is about twenty years old.

I don’t know what is ideal; I think that doctors of medicine are people whose interest in their patients dominates over their other interests. Doctors have to devote themselves entirely to their patients. My father taught me this, and this is how I continue to see medicine. Everything a doctor does, including research, must be done in the patient’s interest, because in the end the results of our research influence treatment. The patient is the doctor’s primary interest. I think that a doctor is first of all a human being, and not only a human being, but also a humanist. It is not enough for a doctor merely to prescribe medication; a doctor must have empathy, must have compassion and has to understand their patients’ emotions. If one has such a personality, they can become a good doctor. If not, someone can study medicine and know the subject perfectly, but they should become a scientist or go into some other profession; they should not be a doctor.

My father was a pediatrician but when World War II began, he was sent to the front and became a military doctor. For people in the Soviet Union, World War II began in 1941 and was called the Great Patriotic War. My father remained at the front until 1945 and he was not discharged when the War ended. He was in the army for a total of ten years. He was a great man, and my mother also was a great person.

It is difficult for me to talk about patient-physician relationships. Since we work with patients with kidney diseases, especially patients who have undergone kidney transplantation, the relationship is a personal one, more complex than most patient-physician interactions. We deal with patients with chronic diseases; we follow them for many years and learn all about their lives, families etc. For example, young women after kidney transplantation may seek our advice about getting married, having children and so on… We advise these women to return to normal life and help them to do this. We keep in mind that before, during and after surgery they risked dying. Dialysis creates a great deal of psychological stress and some patients lose their jobs, friends and even families. Often they are young people who are not yet married. Due to their condition, due to the fact that they faced death, their thinking differs from the thinking of healthy people. As a result, their social network changes; they need to find work and we often help them to adapt socially. Many of our patients work in our center as technicians, for example.

While medical practice in western countries generally includes providing psychological and social follow-up, this is not routine in Russia. We have very few psychologists and social workers working with us, so in fact we provide psychological and social support ourselves. To some extent the doctor has to provide psychological assistance and help patients to solve social issues. Often we even have to help our patients to find the money to pay for medication. I do not think it is a weakness; on the contrary, I think it is fully functional aid. Just imagine – you are visiting a doctor as a patient and the doctor says in a cool voice: you have cancer; you should write your will; goodbye, have a nice day. I don’t like this style, which perhaps corresponds to another lifestyle. Of course, this is my personal point of view and of course other people have different ones. Especially now that we have a wide network of private medical facilities many doctors have become more aloof from their patients. This depends on your personality, and again my view is that as a doctor, you should first of all care about your patients. If you are able to console your patients, you will work much more actively and effectively and your patients will trust you. If you are confident about your knowledge and your patients see that you really want to help them, they are more likely to follow your prescriptions and advice. As a result, treatment will be more effective. If instead you just tell a patient that he or she should not eat this or that, without any attempt to personalize your advice, the patient will probably go and see another doctor. And if you explain why what you are recommending is important, perhaps give a patient some information about blood pressure or how the kidneys work, they will understand and trust you and follow your advice.

I will tell about you one episode from my practice. I had a patient, a young woman with lupus. She needed steroids bur refused to take them. I had to struggle with her every day to try to get her to take her pills. She took her steroids very irregularly and as a result her condition worsened, and her psychological status worsened as well, so she did not eat and did not even drink water. We thought she was in a coma and started her on IV steroid treatment, but she did not improve. One day, her mother came and said we should stop treatment because it was dangerous and could give rise to complications. She also said that her second daughter, who was taking care of her sister, was in danger due to the heavy physical and psychological burden care involved. She said we should let our patient die to avoid harming her sister. I said “No!” and she answered that if there were complications she would take me to court. I told her she could do whatever she wanted to. After this, our patient improved dramatically and one of the first things she did was to refuse to let her mother visit her. I think that when we thought she was in a coma, she was actually severely depressed. Her mother and I spoke in the corridor but I think she heard the conversation. Things happen. Later the mother and daughter made peace and even invited me to visit them at home.

The most disastrous situations were due to lack of dialysis. I can share with you a situation when I made a mistake. We had a patient, a journalist, with the ESRD who needed dialysis. There seemed to be no emergency, no difference whether she started dialysis immediately or two weeks later. Another patient, who urgently needed to start dialysis, was admitted and my mistake was that I talked to the first patient, who seemed to be a reasonable, intelligent person, explaining to her that we had just one place for dialysis, and would start dialysis for another person whose condition was much worse than hers, and she would be taken next. She became furious and accused me of trying to kill her. After this case I realized that neither educational level nor professional skills are important when one’s life is involved. People (of course there are exceptions) don’t care about others’ lives when their own life is in danger. Coronary patients, for example, generally are not very upset when a fellow patient in the ward dies.

The lack of dialysis is still our principal problem. This is because for many years, as I said, renal replacement therapy was ignored by the medical authorities. In the last 15 years there has been rapid progress, but because we lack adequate financing, the problem has not yet been solved. For example Moscow and Saint Petersburg have good dialysis coverage compared to other regions, but we still need twice the number of places we currently have. There is a program of developing dialysis treatment, and I hope that within 5 years or so the problem will no longer exist.

I had a chance to meet some of the world’s leading nephrologists in Denver last year when Robert Schrier organized a special meeting of ISN leaders in frame of ASN, to celebrate 50th anniversary of ISN funding. The ISN leaders told the story of how ISN had become interested in the development of nephrology around the world. One of them recalled his visit to Africa many years ago: he and his colleagues were so shocked that they decided to impact the development of nephrology everywhere. I wondered why these very wealthy people (Professor Schrier’s house looked like a museum) cared about Africa, but they do care and they want to help. There are many people who are ready to give their time and knowledge to improve our world.

Who can Russia teach and what can we teach them? I don’t know. In the field of nephrology, it is the question to ask. Different countries, different people, different experience. In my view communication is important: if you can pick up something, you will. It’s important to share experience, not only professional but also life experience – our attitudes, our perceptions. It is very difficult to characterize a country in a few words… Russian history is complicated, and the twentieth century was really tragic. My parents, for example, lived through three wars. Three wars! Both died at the age of 63. Many different things can be taught. During the Great Patriotic War people helped each other and almost everybody lived in friendship. Nowadays instead, people are angry because of the huge disparities in wealth that exist. I think that communication is vitally important. When the representatives of different nations communicate they enrich each other by transmitting feelings, knowledge, spiritual experience etc.

Since the end of the nineteenth century, Russian medicine has been characterized by humanism. All the best known Russian doctors were humanists and the schools they established were humanistic. The times we live in are different. The political regime is also quite different and our system is now bureaucratic and oligarchic. As a result there is a huge economic gap with enormous differences in living standards. Now some doctors’ principal concern is to make as much money as they can. Interestingly, most of these doctors work in private institutions, while the majority, who are still first of all humanists, work in government institutions.

**Fig. 2:**
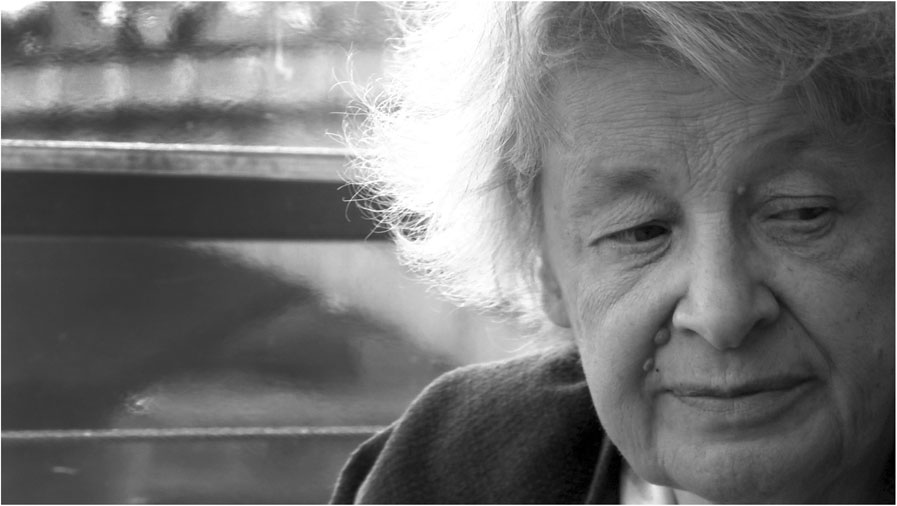
Natalia Tomilina in Prague, June 2011

And now we come to a difficult question. During my lifetime, my views and attitudes have changed. Of course I love my family but I don’t want to talk about them. You know, in the Soviet Union women had to work. I think that a woman should live a full-fledged life, everything should be in a harmony, and a woman should be just a woman at least some of the time. Nevertheless she should have her own position in the life. I love truth and fairness and I detest lies. Well, not to be sentimental – my life is full of music. My husband and I love music, and about once a month we have concerts at home with friends of ours who are recognized musicians playing classical music. Giuseppe Remuzzi attended one of these concerts. Many years ago I enjoyed travelling and mountain climbing, well, many things…. I still like to travel. My husband and I are in love with Italy, Florence in particular. I even have a very special feeling if something reminds me of Florence. Once, we were in Paris and our hotel was next door to a nineteenth-century palace, whose name I forget, where Italian masterpieces are exhibited – Donatello and others. You enter and feel as though you are in Florence. My dream is to visit Florence again (Fig. 2).

What I definitely like – I like to teach. As for the poetry of our profession – I don’t know. Being a doctor, I see that our work is hard and demands physical and emotional efforts. We are sitting here now, and there is a lot of poetry around, but our work is often heart-breaking. When your patient is in critical condition, there is nothing poetic about that. You may have extremely severe patient, whom you managed to save, and he leaves the hospital without even saying “thank you”. It happens. Instead, you might do nothing, only smile and give advice, and the patient feels very grateful. We do not work for our patients’ gratitude or because of the poetry. We work because it is our job.

As you gain professional experience you become passionate about your work. You get a severe case, and you passionately want to make a diagnosis, to treat the patient properly and save a life. For example, we admitted a patient with thrombotic thrombocytopenic purpura. She was in a coma, but we saved her – we were exceptionally lucky, I presume. Or you rescue a patient who is clinically dead, which also involves luck. The patient may not realize that they could have died and does not feel grateful. Well, let it be. Once, when I was a child, my father explained to me that being a doctor involves passion. He said you could get a crying, sick infant, who could not even speak, and you had to understand its condition, its illness, and comfort the baby. This is real passion, in some sense a hazard, and I recall his words often.

What advice would I give to someone who wants to become a doctor? Well, to become a good doctor you need knowledge. Therefore my advice is to study hard. To study more, to study in depth, to go beyond the textbooks; otherwise it is impossible to become a good doctor. Secondly, it is necessary to train what I call your “emotional imagination”. An ability to imagine the feelings of a patient is, in my opinion very important. And you have to love your profession. Usually people like what they are good at. Someone who finds it difficult to add and subtract will not like mathematics. If one does not love practicing medicine, it is better not to become a doctor. If somebody really wants something, they can achieve their goal. If someone just wants something but does nothing to achieve it that is senseless.

**Fig. 3:**
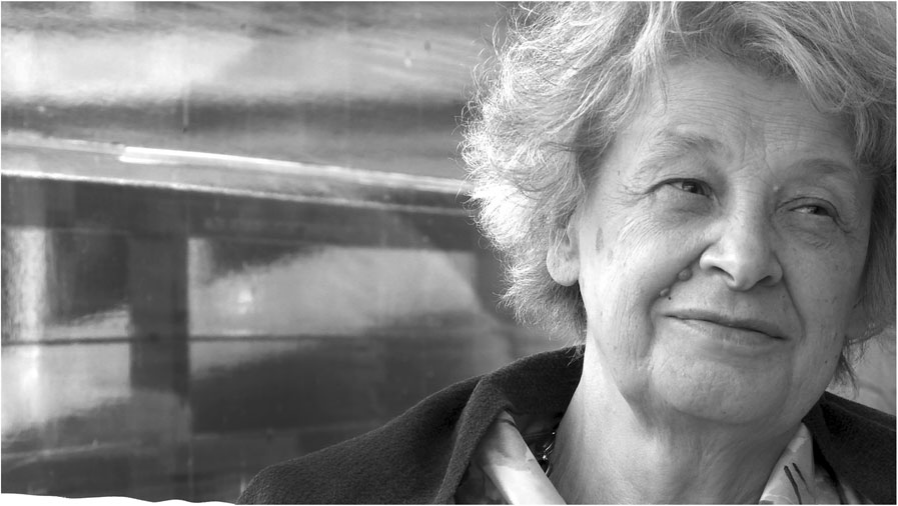
Natalia Tomilina in Prague, June 2011

I’ve said a lot…I’ve always thought that my position was very clear, but I find it is not easy to express myself. It is not easy to answer some of the questions you asked. I’ve done my best (Fig. 3).

